# Feasibility of a Hospital at Home for Management of Acute Severe Ulcerative Colitis: A Retrospective Case Series

**DOI:** 10.1155/crgm/4355773

**Published:** 2025-08-18

**Authors:** Anupama A. Goyal, Jeffrey A. Berinstein, Bishu Shrinivas, Peter D. R. Higgins, Stephanie Taylor

**Affiliations:** ^1^Division of Hospital Medicine, University of Michigan, Ann Arbor, Michigan, USA; ^2^Division of Gastroenterology, University of Michigan, Ann Arbor, Michigan, USA

## Abstract

The Centers for Medicare and Medicaid's Acute Hospital Care at Home waiver in 2020 has enabled the management of acute conditions that were traditionally cared for in the hospital to transition to the home setting. To our knowledge, data regarding the management of patients with acute severe ulcerative colitis (ASUC) in hospital at home (HaH) programs has not been reported. We conducted a retrospective review of ASUC patients admitted to our HaH program from our adult hospital, who demonstrated early clinical response but required a comprehensive, closely monitored treatment environment that was provided in the patients' homes. Patients received daily evaluations by hospitalists, gastroenterologists, and registered nurses with clinical assessments, monitoring of vitals, and therapeutics (e.g., intravenous fluids, corticosteroids, etc.), alongside daily blood monitoring for worsening inflammation. Patient demographics and UC disease characteristics were extracted from electronic health records. Outcomes of interest included emergency department (ED) visit, readmission, and mortality within 30 days of index admission; hospital-acquired conditions (HACs) of interest (delirium, catheter associated infections, and falls) during HaH stay; length of stay (LOS) in traditional hospital vs. HaH phase; and need for escalation back to traditional hospital. Three eligible and consenting ASUC patients were transferred to HaH. Two were female and one male, with a mean age of 59.7 years. The mean LOS in the traditional hospital was 8.7 days (range: 4–18), and 6 days (range: 4–9) in HaH. There were no escalations from HaH back to the traditional hospital. One patient had a 30-day ED visit (33%) with readmission (33%). There were no deaths within 30 days of index admission or documented HACs during HaH stay. Our case series highlights the preliminary feasibility of HaH for the management of ASUC patients, as a promising alternative to prolonged hospital-based care, without compromising patient safety or care quality.

## 1. Introduction

Acute severe ulcerative colitis (ASUC), a medical emergency requiring hospitalization, affects about one-fifth of UC patients with high healthcare utilization [1, 2]. Hospitalizations for ASUC can exacerbate psychologic distress and underlying anxiety that negatively impact patients' quality of life [3].

Hospital at home (HaH) programs have enabled management of traditionally hospitalized acute conditions to transition to the home setting. Data demonstrate that patients who received care in HaH have low rates of mortality with fewer hospital-acquired conditions (HACs) (e.g., falls), along with lower costs of care. Furthermore, patients in HaH reported positive experiences, promoting healing (e.g., better sleep), and improved quality of life [4, 5].

The acute diagnoses being managed in HaH are expanding beyond infections to include postoperative and malignant conditions, without compromising patient safety [6]. Data regarding the management of patients with ASUC in HaH have not been reported. The aim of this case series is to describe and assess the feasibility of an innovative care pathway for treating ASUC through our HaH program.

## 2. Method

**Figure 1:**
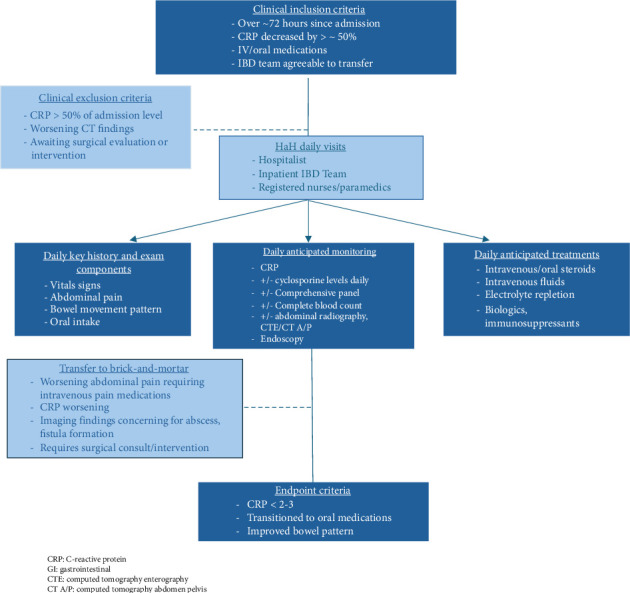
Acute severe ulcerative colitis hospital at home pathway. University of Michigan severe ulcerative colitis protocol: https://www.med.umich.edu/ibd/docs/severeucprotocol.pdf.

We conducted a retrospective review of ASUC patients admitted to the University of Michigan Hospital between May and August 2024. Our ASUC HaH pathway offered home-based care for patients with an episode of ASUC, as defined by Truelove and Witts' Criteria, demonstrating early clinical response yet requiring further hospital-level monitoring and therapy (Figure 1) [7]. Patients with ASUC transferred to HaH continued to receive monitoring according to our institutional Severe Ulcerative Colitis Protocol [8]. All required equipment to provide care for the HaH patients, such as patient monitoring devices (e.g., blood pressure machine, pulse oximetry, weighing scale, and thermometer) and telemedicine devices (e.g., audiovisual tablet, backup battery, and Wi-Fi, with technician availability 24/7 to address any connectivity issues), were delivered to patients' homes. Once transferred to HaH, ASUC patients received daily virtual evaluation by a hospitalist with an in-person registered nurse or community paramedic, as well as daily virtual consultation with the gastroenterology team. The institution's secure electronic health record systems were used for record keeping and order entries (such as medications and tests/imaging/consultations). Anticipated therapies available through HaH include IV/oral corticosteroids, daily/as needed laboratory and imaging tests (e.g., X-rays in home and CT scan at hospital department), fluid and electrolyte replacement, and initiation and monitoring of rescue therapies if required (infliximab, cyclosporine, tofacitinib, or upadacitinib). As per protocol, patients experiencing clinical deterioration marked by consistently rising CRP, escalating pain or bowel symptoms, concerning imaging, or the need for in-person surgical consultation or immediate surgical intervention were rapidly transferred back to inpatient care within 2–4 hours.

Eligible patients signed a consent for treatment in HaH form approved by our institutional Office of General Counsel. Patient demographics and UC disease characteristics were extracted from electronic health records. Outcomes of interest included emergency department (ED) visit, readmission, and mortality within 30 days of index admission; HACs of interest (delirium, catheter-associated infections, and falls) during HaH stay; length of stay (LOS) in traditional hospital vs. HaH phase; and need for escalation back to traditional hospital. Data were presented descriptively. Patients were deemed stable for discharge from HaH when they demonstrated clinical improvement and CRP was < 2-3.

## 3. Results

Three eligible and consenting ASUC patients were transferred to HaH. Two were female and one male, with a mean age of 59.7 years (Table1). The mean LOS in the traditional hospital was 8.7 days (range: 4–18) and 6 days (range: 4–9) in HaH. There were no escalations from HaH back to the traditional hospital. One patient had a 30-day ED visit (33%) with readmission (33%). There were no deaths within 30 days of index admission or documented HACs during HaH (Table 2).

### 3.1. Case 1

A 36-year-old male with a 19-year history of UC, in clinical remission off all maintenance therapy, was admitted with > 10 bloody bowel movements/day and abdominal pain. Admission CRP was 21.2 mg/dL (mg/dL). Endoscopy demonstrated Mayo 3 disease. He was treated with intravenous (IV) methylprednisolone 30 mg and upadacitinib 30 mg, both twice daily. The decision was made to continue medical management jointly with colorectal surgery. Patient had a prolonged 18-day stay in the physical hospital, as he had a very slow normalization in CRP with a rapid uptick in CRP to 2.4 mg/dL on attempt at transitioning off IV corticosteroids. His upadacitinib was discontinued, and cyclosporine 2 mg/kg/day was initiated for 7 days. His CRP decreased to 0.7 mg/dL, with 4 bowel movements/day with minimal blood, and he was transferred to HaH on oral prednisone 60 mg daily and cyclosporine 250 mg twice daily, due to the high-risk nature of the patient's disease and the need to confirm the durability of improvement, for day-to-day cyclosporine dose adjustments and to monitor and correct for electrolyte derangements from his cyclosporine. Ultimately, he spent 5 days in HaH to complete this close monitoring with < 4 bowel movements/day with no blood. No ED visit, readmission, or mortality were reported within 30 days of admission. No HACs were documented during HaH.

### 3.2. Case 2

A 70-year-old female with a 6-year history of UC, on maintenance mesalamine, as well as 7 days of prednisone 40 mg, was admitted with abdominal pain, 6-10 bloody bowel movements/day, and CRP of 8.7 mg/dL. Endoscopy demonstrated Mayo 3 disease, and she was treated with IV methylprednisolone 30 mg twice daily. Despite 48 h of treatment, CRP increased to 8.9 mg/dL with worsening clinical symptoms. She was initiated on infliximab 10 mg/kg over 4 days with clinical improvement. On transfer to HaH, CRP was 3.3 mg/dL, and she reported 2-3 soft nonbloody bowel movements/day. Her CRP decreased to 2.1 mg/dL and then up trended to 2.9 mg/dL. She underwent a sigmoidoscopy in the hospital procedure unit that demonstrated Mayo 2 disease. Postprocedure, she returned to HaH. After spending 4 days total in HaH, she was discharged with CRP of 2.7 mg/dL and 1-2 soft nonbloody bowel movements/day, on prednisone taper, mesalamine enema, and cortisol enema for 2 weeks with outpatient infliximab induction. The patient experienced one ED visit with admission within 15 days of discharge unrelated to ASUC. No HACs were documented during HaH.

### 3.3. Case 3

A 73-year-old female with an 8-year history of UC, on infliximab, was admitted for > 6 bloody bowel movements/day and abdominal pain with CRP of 23.5 mg/dL. Endoscopy demonstrated Mayo 3 disease. She was initiated on IV methylprednisolone 30 mg twice daily. The decision was made to continue medical management jointly with Colorectal Surgery. Due to a lack of sufficient clinical improvement on corticosteroids, the patient received an additional infusion of infliximab 10 mg/kg as it was suspected that the patient was receiving insufficient infliximab dosing prior to admission. CRP was 3.9 mg/dL on transfer to HaH with 1-2 soft nonbloody bowel movements/day. The patient spent 9 days in HaH with continued clinical improvement. CRP was 1.8 mg/dL on discharge from HaH with 1 solid nonbloody bowel movement/day, on oral prednisone and outpatient infliximab infusions. No ED visit, readmission or mortality were reported within 30 days of admission. No HACs were documented during HaH.

## 4. Discussion

Our case series of eligible ASUC patients treated in our HaH program demonstrates the preliminary feasibility of HaH for the management of ASUC. The 30-day mortality rate was zero. Patients did not have any documented HACs (i.e., falls, delirium, or catheter-associated infections) during their HaH stay. One patient underwent a sigmoidoscopy in the traditional hospital procedure unit directly from HaH without requiring the use of an inpatient bed. No patients required escalation of care back to our traditional hospital for medical management. One patient experienced an ED visit and subsequent admission within 30 days of admission, for a respiratory infection, possibly related to their ASUC immunosuppressive therapy, which was rapidly identified and treated.

Management of ASUC in the home setting is not a standard practice in the United States [9]. A study in the United Kingdom of ASUC patients reported no differences in the primary outcome of colectomy during ASUC between inpatient (15%) vs. ambulatory (13%) vs. inpatient and then discharged to ambulatory (13%) settings [10]. A qualitative analysis found that ASUC patients were highly motivated to avoid hospital admissions or even shorten inpatient stays, suggesting that patients may be willing to undertake intensive home-based care to maintain comfort and proximity to their family [11]. The implementation of a HaH pathway for ASUC patients presents a promising alternative to prolonged hospital-based care while maintaining continuity of treatment in a supervised, home setting, with the potential to address ASUC patients' unmet need to get care at home.

Our hypothesis-generating results should be interpreted in the context of several limitations. We did not collect data on the number of patients who met eligibility criteria, were approached, and consented (or declined), information that would be helpful to understand what factors influence (or not) a patient's acceptance of HaH. Furthermore, we did not collect patient experience data to understand if the care delivery model of HaH is preferable to the traditional model. Importantly, our patients transitioned to HaH after initial management and improvement in a brick-and-mortar setting, limiting the generalizability of our findings to full treatment of ASUC in HaH. In this preliminary phase of the HaH pathways, we enrolled only ASUC patients who had a clear robust response to therapy administered in the hospital. As our HaH program gains further experience and we build confidence in our ASUC patient selection, we hope to safely broaden eligibility to include additional higher-risk patients exhibiting early clinical signs of clinical improvement.

## 5. Conclusion

Our experience with the ASUC HaH pathway demonstrates promising feasibility, offering a potential model for high-acuity home-based care that alleviates the burden on hospitals without compromising care quality or patient safety. In this preliminary phase of the HaH pathways, we enrolled only ASUC patients who had a clear robust response to therapy administered in the hospital. As our HaH program gains further experience and we build confidence in our ASUC patient selection, we hope to safely broaden eligibility to include additional higher-risk patients exhibiting early clinical signs of clinical improvement. Larger, prospective studies are also needed to validate and refine the HaH model for ASUC on a broader scale.

## Figures and Tables

**Table 1:** Patient and disease characteristics.

Patient age/sex/race	Traditional hospital admission CRP (mg/dL)	Traditional hospital admission endoscopic Mayo score	Traditional hospital admission albumin (g/dL)	CRP at the time of transfer to HaH (mg/dL)	ASUC therapeutics at the time of transfer to HaH	Discharge CRP (mg/dL)
36/Male/Caucasian	21.2	Mayo 3	3.1	0.7	IV Methylprednisolone, cyclosporine	0.7
70/Female/Caucasian	8.7	Mayo 3	3.2	3.3	IV Methylprednisolone and infliximab × 2 doses	2.7
73/Female/Caucasian	23.5	Mayo 3	4.1	7.1	IV Methylprednisolone and infliximab × 1 dose	1.1

**Table 2:** Patient-related outcomes.

Patient age/sex	LOS in a traditional hospital	LOS in HaH	Escalation back to the traditional hospital	30-day readmission	30-day ED visit	30-day mortality	Reported hospital-acquired conditions
36/Male	18	5	No	None	None	No	None
70/Female	4	4	No	Yes	Yes	No	None
73/Male	4	9	No	None	None	No	None

Abbreviations: ASUC, acute severe ulcerative colitis; CRP, C- reactive protein; HaH, hospital at home; LOS, length of stay.

## Data Availability

Data sharing is not applicable to this article as no new data were created or analyzed in this study.
